# Lipid Oxidation in Emulsions Fortified with Iron-Loaded Alginate Beads

**DOI:** 10.3390/foods8090361

**Published:** 2019-08-24

**Authors:** Alime Cengiz, Karin Schroën, Claire Berton-Carabin

**Affiliations:** 1 Food Engineering Department, Engineering Faculty, Ondokuz Mayis University, Samsun 55139, Turkey; 2 Food Process Engineering Group, Wageningen University, Bornse Weilanden 9, 6708WG Wageningen, The Netherlands

**Keywords:** Iron encapsulation, iron fortification, ferrous sulfate, alginate beads, ionic gelation, O/W emulsion, lipid oxidation

## Abstract

The potential use of iron-loaded alginate beads to fortify oil-in-water (O/W) emulsions was studied. Iron-loaded alginate beads with different sizes (0.65, 0.84, 1.5 and 2 mm) were produced by ionic gelation with calcium chloride, leading to 81% encapsulation efficiency (EE) of ferrous sulfate. These beads were added to O/W emulsions to investigate their effect on lipid oxidation. The use of iron-loaded alginate beads inhibited lipid oxidation in emulsions, compared to a control emulsion with the same concentration of free ferrous sulfate in the continuous phase, but did not totally prevent it. Results obtained with scanning electron microscopy and energy dispersive X-ray spectroscopy (EDX) analysis showed that some reactive iron was present at the surface of the beads. Oxidation of the lipid droplets was slightly higher for smaller alginate beads, suggesting that the reaction could be linked to the total bead surface. When covering iron-loaded beads with an extra layer of alginate, lipid oxidation was inhibited, which confirmed the role of reactive surface-bound iron. This study shows that the location of iron within the encapsulates plays a crucial role in the chemical stability of fortified foods and should be taken as a starting point in the design of iron-fortified food products.

## 1. Introduction

Iron deficiency anemia is a worldwide health problem, for which the fortification of foods with iron is the most cost-effective prevention strategy [1]. However, iron is a challenging micronutrient; unpleasant taste characteristics and the acceleration of oxidative reactions are major consequences of the direct incorporation of iron in food products [2]. Various attempts have been made to keep iron apart from oxidizable substrates; in previous work, for example, we used liposomes, but these were ineffective because the phospholipids became oxidized and thereby contributed to the reaction’s propagation [3]. It was thus clear that alternative strategies should be considered, for example the use of alginate beads. 

Over the years, alginate hydrogels have been successfully used for the encapsulation of numerous food ingredients such as probiotics [4], lipids [5], enzymes [6], and antioxidants [7] to prevent interactions with other food components, or gastric fluid. In this work, we use a similar approach, trying to segregate iron from its reactants via physical encapsulation in an alginate hydrogel, and thus limit or prevent its pro-oxidant activity. 

Alginate is a natural anionic polysaccharide typically obtained from brown seaweed (*Phaeophyceae*) [8]. Because of its non-toxicity, low cost, biocompatibility and mild gelation properties, the use of alginate has a wide range of applications such as in encapsulation for food, pharmaceutical, biomedical, and agricultural pest control materials [9]. Alginate gel can be formed by the ionic-crosslinking of guluronic acid residues (G–G sequences) in the presence of multivalent cations (e.g., Ca^+2^, Ba^+2^, Fe^+2^, Fe^+3^, Al^+3^) and forms a so-called “egg box” structure, whereas classical gels are held together by van der Waals forces. Since the beads have a considerable size, the process of ionic gelation starts at the outer layers of the bead, and continues to the center through the diffusion of the metal ions, which takes some time [10]; depending on the size of the beads and the concentration of ions used, this is typically in the order of minutes to even hours. It is expected that iron can thus be bound inside the alginate beads to limit its interactions with the outside environment, such as oil droplets in food systems [9]. 

Ionic gelation has been successfully used to encapsulate soluble iron in alginate beads [11,12,13,14,15,16,17,18,19] at a high encapsulation efficiency for different iron sources [14,15,17]. An advantage is also that such systems can lead to high iron release in the duodenum, where iron absorption takes place, because of the physical disintegration of the alginate beads in duodenal conditions [13,14,16,19]. The actual application of such beads has been reported for only a limited number of food applications, such as milk [11], probiotic yogurt [12], and cocoa-containing nut paste [20]. In milk enriched with iron-loaded alginate beads, no oxidation-related sensory defect was found; in yogurt and nut paste, entrapping iron in alginate beads retarded lipid oxidation over regular shelf-life tests, compared to products with free iron, but did not fully prevent it.

From the above, it is clear that alginate beads are promising vehicles for iron encapsulation but, in order to prevent undesired lipid oxidation, more mechanistic information is needed. The purpose of the present study was to investigate the pro-oxidant activity of iron-loaded alginate beads in oil-in-water (O/W) emulsions, representing model food systems that contain oil droplets. Besides being a convenient measuring system, the sensitivity to oxidative effects allows us to evaluate the efficiency of encapsulation. The oxidative stability of O/W emulsions fortified with iron-loaded alginate beads was compared to that of control emulsions that contained empty alginate beads, or non-encapsulated ferrous sulfate. We systematically varied alginate bead size to modulate the fraction of surface-bound iron, which is potentially the reactive fraction with regard to lipid oxidation. We also successfully mitigated the issue of reactive surface iron by applying a second alginate layer onto the iron-loaded beads. 

## 2. Materials and Methods

### 2.1. Materials

Rapeseed oil purchased from a local supermarket was stripped by means of alumina to eliminate impurities and tocopherols [21]. Sodium alginate (W201502, medium viscosity (5–40 mPa s)), Tween 20, calcium chloride, ferrous sulfate heptahydrate (FeSO_4_, 7H_2_O), alumina powder, para-anisidine, 3-(2-pyridyl)-5,6-di(2-furyl)-1,2,4-triazine-5′,5″-disulfonic acid disodium salt, n-propanol, hexane, L-ascorbic acid, sodium acetate trihydrate, and acetic acid were purchased from Sigma Aldrich (St. Louis, MO, USA). Ultrapure water, purified by a Millipore Milli-Q system (Darmstadt, Germany), was used throughout the study.

### 2.2. Preparation of Alginate Beads

Ionically cross-linked alginate hydrogel beads were produced through the injection method shown in the supplementary information (Figure S1). An alginate solution was prepared by mixing 3 g alginate and 90 g ultrapure water, followed by gentle stirring for 2 h at room temperature. One gram ferrous sulfate was dissolved separately in 9 g ultrapure water, and the solution formed was added to the alginate solution to reach final concentrations of 30 g/L alginate and 10 g/L ferrous sulfate, which is far below the solubility limit of the latter in water, ~250 g/L [22]. The mixture was stirred at 400 rpm for 15 min. To remove air bubbles, the mixture was placed in an ultrasonic bath for 10 min. After that, 10 mL alginate-iron mixture was added dropwise with a syringe pump at 1.0 mL min^−1^ into 25 mL of the hardening bath (5 wt% CaCl_2_) that was kept under magnetic stirring. The nozzle tip (inner diameter of 0.41 mm: Nordson EFD, Alcester, UK.) was positioned 6–7 cm above the bath. 

The bead size was controlled through the air pressure, and 0.2, 1, 2, and 3 bar were used to produce very large- (VL), large- (L), medium- (M) and small- (S) sized beads, respectively. After production, the beads were stirred in the hardening bath at room temperature for 30 min to allow crosslinking with Ca^2+^, and subsequently stored in the same CaCl_2_ solution overnight at 4 °C to form strongly gelled beads. All beads were stored in the fridge in CaCl_2_ solution until use. Before further use, the beads were collected from the CaCl_2_ solution and washed with ultrapure water for 10 min to remove unbound Ca^2+^ ions and free Fe^2+^ irons, after which the beads were filtered using filter paper (Whatman 41). Some alginate beads were also produced without ferrous sulfate (referred to as empty beads) and used for reference experiments.

The bead size was determined by static light scattering (Malvern Mastersizer 3000, Malvern Instruments Ltd., Malvern, Worcestershire, UK), using refractive indices of 1.33 and 1.47 for water and beads, respectively. The obscuration was kept between 4 and 7%.

For the production of double beads, very large (VL) beads were used as a starting point and immersed in a 1% alginate solution for 30 min to allow for an extra alginate coverage, after which they were transferred into a 5% CaCl_2_ solution to harden for 30 min. Again, these beads were stored in the fridge in a CaCl_2_ solution until use.

### 2.3. Encapsulation Efficiency (EE) of Iron-Loaded Alginate Beads

The iron content of alginate beads was determined by the spectrophotometric ferene method [3]. In brief, ferrous iron reacts with the chromogen ferene to form a blue chromophore, and the intensity of the color is proportional to the amount of iron present in solution. 

To measure ferrous iron, a dissociation agent is used, which is a mixture of ascorbic acid (0.25 M) and acetate buffer (1.4 M, pH 4.5). This mixture was added to the sample (liquid phase surrounding the beads) and mixed with ferene solution (6 mM). After 5 min, the absorbance at 593 nm was measured. A calibration curve was used to determine the concentration. 

The amount of free iron ($Fe_{f}$), measured in the liquid phase surrounding the beads, and the total amount of iron ($Fe_{T}$) in alginate solution were used to calculate the encapsulation efficiency (EE) as shown in Equation (1):

(1) $$\mathrm{EE}\;(\%)=(Fe_{T}-Fe_{f})/Fe_{T}*100$$

### 2.4. Morphological Characterization of Alginate Beads

The surface morphology of medium-sized alginate beads was examined using scanning electron microscopy (SEM) and the elemental composition of the bead surface was determined using energy-dispersive X-ray spectroscopy with a field emission scanning electron microscope (FEI Magellan 400/Oxford Instruments, Hillsboro, Oregon, USA).

Sample preparation was as follows: alginate beads were cleaned with ultrapure water, filtered and air-dried at 40 °C for 90 min. Next, the samples were mounted on metal stubs, using double-sided adhesive tape, coated with a layer of gold under vacuum, and then examined at 2 kV. The experiments were performed twice using independent batches.

### 2.5. Preparation and Physical Characterization of Emulsions

*Emulsion preparation.* Tween 20 solution (1 wt%) was added to ultrapure water and stirred overnight at room temperature. A primary emulsion was prepared by homogenizing 10 wt% stripped rapeseed oil with 90 wt% of the aqueous phase mention earlier using a high-speed rotor stator homogenizer (UltraTurrax T25 Basic Disperser with 25 mm diameter blade, Janke and Kunkel, IKA, Staufen, Germany) at 7000 rpm for 2 min. Subsequently, the primary emulsion was passed through a high-pressure homogenizer (M-110Y Microfluidizer, Microfluidics, USA) equipped with an F12Y chamber at 800 bar, for 3 cycles. To limit temperature rise during the emulsification process, the cooling jacket of the homogenizer was filled with iced water. Emulsion preparation was performed twice to obtain independent duplicates. The procedure for the addition of iron or beads is described in the next section. 

*Particle size distribution.* The emulsion droplet size was determined by static light scattering (Malvern Mastersizer 3000, Malvern Instruments Ltd., Malvern, Worcestershire, UK). The obscuration was between 8 and 12%; the refractive index (RI) of the dispersant was set to 1.33 for water, and to 1.46 for the dispersed rapeseed oil. 

*Zeta potential.* The surface charge (zeta potential) of the emulsion droplets was measured with a dynamic light scattering instrument (Zetasizer Nano ZS, Malvern Instrument Ltd., UK). Samples were 100-fold diluted with ultrapure water prior to measurement. The results are expressed in mV, as the mean value ± standard deviation. 

Physical characterization of the emulsions was performed immediately after emulsification, and at the end of the incubation period. For the samples containing alginate beads, the latter did not interfere with the characterization of the oil droplets, as the beads sedimented to the bottom of the tubes as soon as agitation was stopped, due to their large size.

### 2.6. Measurement of Lipid Oxidation and Hydrolysis

*Incubation conditions.* Emulsions were partitioned in 50-mL tubes (40 g per tube) and analyzed over 8 days. Four different systems were studied: control emulsions (no iron added), emulsions containing iron-loaded alginate beads or empty beads (0.28 g beads per tube) and emulsions containing non-encapsulated (free) ferrous sulfate. For both iron-containing systems, the final iron concentration in the emulsions was 200 µM, which is much lower than the solubility limit of ~1 M. For the emulsion added with free iron, ferrous sulfate was added as dry powder that dissolved instantly upon vigorous mixing. For the emulsions that contained beads, appropriate bead amounts were added, and the samples were quickly mixed by hand. All samples were then incubated at 25 °C, on a rotating agitation device (5 rpm) to prevent creaming/sedimentation and ensure sample homogeneity. 

*Primary lipid oxidation products.* The amount of conjugated diene hydroperoxides was measured using the method described by Lethuaut and co-workers [23]. Briefly, 0.25-mL emulsion aliquots were diluted 100 times in n-propanol, and the mixture was centrifuged at 1200× *g* for 4 min. The absorbance of the supernatant was recorded between 200 and 310 nm using a UV/VIS spectrophotometer (DU 720 Beckman Coulter, Brea, CA, USA). Results were expressed in mmol conjugated diene hydroperoxides per kg of oil (mmol CD kg^−1^ oil) using 27,000 M^−1^ cm^−1^ as the molar extinction coefficient of conjugated dienes at 233 nm.

*Secondary lipid oxidation products.* The para-anisidine value (pAV), which is a global measurement for the formation of aldehydes, was determined by first mixing 0.3 g emulsion with 1.5 mL n-hexane: n-propanol mixture (3:1 *v/v*). The absorbance of the top hexane phase (*Ab*) was measured at 350 nm, using pure hexane as a blank. Then, 1 mL of this top hexane phase was added to 0.2 mL para-anisidine solution (2.5 M in acetic acid) and mixed. After 10 min, the absorbance was measured at 350 nm (*As*), using as blank pure hexane similarly mixed with the para-anisidine solution. The pAV was calculated as shown in Equation (2).

(2) pAV=(1.2As−Ab)/m

where *m* is the mass (g) of oil per mL hexane.

*Acid value.* The acid base titration technique described in the Association of Official Agricultural Chemists (AOAC) Official Method 969.17 [24] was used to determine the formation of free fatty acids. Each oil and emulsion sample was analyzed in triplicate.

### 2.7. Statistical Analysis

Statistical analysis was performed using the SPSS software (version 18, PASW Statistics, New York, NY, USA). One-way analysis of variance (ANOVA) was conducted using six individual results from two independent repetitions and least significant differences were calculated at *p* < 0.05 applying Tukey’s post-hoc test.

## 3. Results and Discussion

### 3.1. Physical Properties of Alginate Beads

The size (Table 1) and encapsulation efficiency (EE) of iron-loaded alginate beads were investigated. The bead size can be well controlled by the applied air pressure, and a range of average sizes was obtained (0.62–2.05 mm). The gel-forming capacity of sodium alginate with selected divalent cations is well documented [9]; e.g., Morch and co-workers [25] showed that Ca^+2^ binds to guluronic acid (G-) and mannuronic-gluronic acid (MG-) blocks, Ba^+2^ to G- and mannuronic acid (M-) blocks, Sr^+2^ to G-blocks only, and Fe^+2^ to G- and M-blocks [26,27,28]. In the presence of both calcium and iron, it is expected that both links that are mentioned for the individual ions may occur, and this could also explain the observed significant difference in size between iron-loaded and empty alginate beads (for example, medium-sized iron-loaded beads were on average 820 µm, while empty beads had an average size of 756 µm).

**Figure 1:**
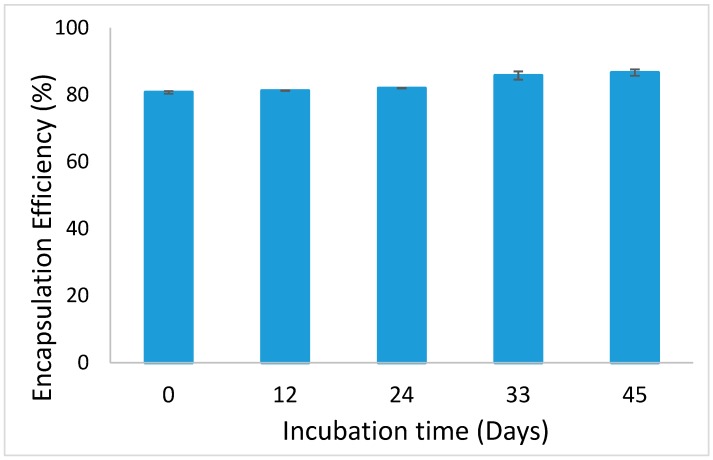
Encapsulation efficiency of medium-sized iron-loaded alginate beads followed in time.

Ferrous sulfate could successfully be encapsulated in alginate beads, with an EE that is approximately 80% (Figure 1). This value did not decrease, and even increased slightly (up to 86%), over 45 days of incubation of the alginate beads, as exemplified for the medium-sized beads in Figure 1. This shows that no iron leaked out from the beads, and it is possible that some iron initially present in the continuous phase may later have adhered to the beads, leading to this increase in the measured EE. In terms of application in products, this is an important feature, since the beads could be stored for considerable time without encapsulation efficiency being affected. 

**Figure 2:**
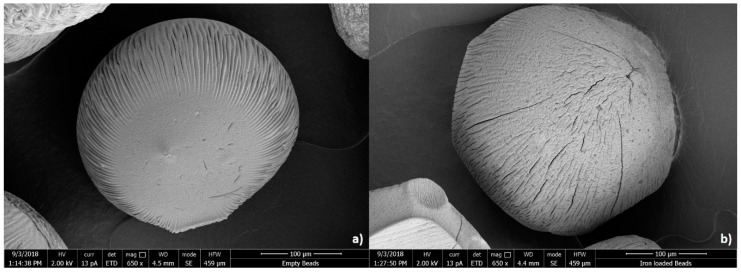
Scanning electron microscopy images of medium-sized (**a**) empty and (**b**) iron-loaded beads that were both air-dried before analysis and are thus smaller than their original size. The scale bar represents 100 µm.

The surface morphology of dried empty and iron-loaded beads was investigated (Figure 2). In line with their macroscopic appearance observed with light microscopy, both beads looked spherical, albeit that the size reduced considerably because of the drying procedure used. SEM analysis showed that iron-loaded beads seemed to have a rougher surface with deeper wrinkles scattered over the entire surface, compared to empty beads, and this was the case for all sizes. 

### 3.2. Physical Properties and Free Fatty Acid Formation in Incubated Emulsions

**Figure 3:**
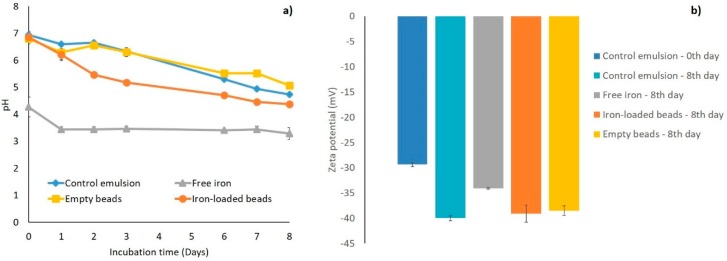
(**a**) pH of the control emulsion (♦) and of emulsions with free iron (▲), iron-loaded alginate beads (●), and empty alginate beads (■); (**b**) Zeta potential of the control emulsion at day 0 and 8, and of emulsions with free iron, iron-loaded alginate beads, and empty alginate beads, after 8 days of incubation. All emulsions were incubated at 25 °C under slow rotative agitation. In iron-containing emulsions, the total FeSO_4_ concentration was 200 µM. Error bars represent standard deviations (*n* = 4).

The droplet size distributions of Tween 20-stabilized emulsions, freshly prepared and after 8 days of incubation, are presented in Figure S2. For all emulsions, monomodal droplet size distributions with an average volume mean diameter (*d_4,3_*) of approximately 130 nm were found, irrespective of the incubation time and presence of alginate beads and/or iron. This indicates that all tested emulsions were physically stable. The starting pH of the iron-free emulsions was approximately 7.0, whereas the emulsion containing non-encapsulated ferrous sulfate had an initial pH of 4.0, that decreased further in time (Figure 3a). It is well documented that metal ions form metal–aquo complexes in water, and these are acidic owing to the ionization of protons from the water ligands, which explains the low pH of the solution [29] 

The pH of the control emulsion and of the emulsion containing empty beads decreased from 7.0 to 5.0 over 8 days of incubation; in the emulsion containing iron-loaded alginate beads, the pH decreased slightly faster and to a higher extent. The fact that the pH of the emulsions containing the iron-loaded alginate beads was higher is in line with the high EE reported earlier, which prevents iron from leaking out and thus prevents the formation of metal–aquo complexes. Thus, the decrease in pH over time observed for all emulsions is probably not linked to any iron release or iron-based chemical reaction, as it was also observed for the emulsions that did not contain iron. Therefore, other chemical reactions may be involved, which we discuss later.

The zeta potential of all emulsions was negative, even though we used a non-ionic surfactant (Figure 3b). The zeta potential reflects the charged species present in the Stern layer surrounding the droplets and has already been reported to have negative values for polysorbate-stabilized emulsion droplets [21]. The fact that the pH is changing as a function of time is an indication that some reactions occur, and that may also affect the zeta potential. Interestingly, as was also noticed for the changes in pH, the changes in zeta potential over the incubation period do not seem to be linked to the presence and physical state of the ferrous sulfate.

It is known that free fatty acids can be formed through triglyceride hydrolysis [30], which decreases the pH. Free fatty acids, especially in their deprotonated form, are surface-active and can decrease the zeta potential of emulsion droplets [31]. This is why we determined the free acidity in our samples (Table 2). Stripped rapeseed oil had a low amount of free fatty acids, 0.02 mg/g oil, which was also the case for the control emulsion. After 8 days of incubation at 25 °C, the free fatty acid content of all emulsions increased, with the control emulsion having the lowest value of 0.65 mg/g oil, whereas both iron-containing emulsions had similar values of approximately 1 mg/g oil. Herman and Groves (1993) [30] also detected free fatty acid formation in emulsions due to triglyceride hydrolysis (and in their case, also of phospholipids), which also happened in our emulsions. Given the fact that free fatty acids partition between the water phase, interface and oil phase, this will have influenced the pH and the charge of the interface. Given the notable difference between the control emulsion and the iron-containing emulsions, it is also expected that other chemical mechanisms, such as lipid oxidation, are involved in the observed pH drop, as will be discussed in the following sections. It is also interesting to mention that the solubility of ferrous iron is increased at low pH [18], which could influence its availability for chemical reactions.

### 3.3. Lipid Oxidation in the Incubated Emulsions

**Figure 4:**
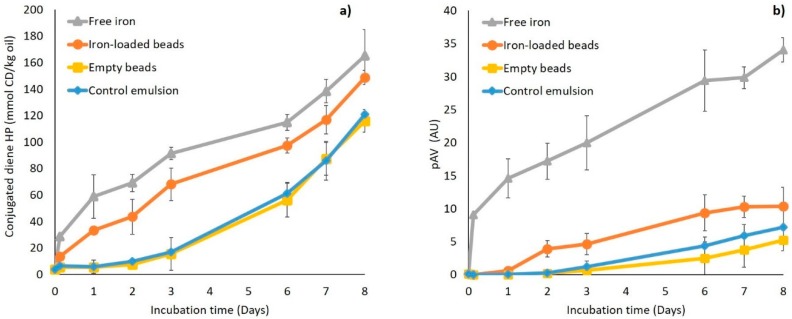
(**a**) Conjugated diene (CD) hydroperoxide concentrations and (**b**) para-anisidine value (pAV) in emulsions containing free iron (▲), iron-loaded alginate beads (●), or empty alginate beads (■), and in the control emulsion (♦). All emulsions were incubated at 25 °C under slow rotative agitation. For the two emulsions containing iron (FeSO_4_), the total concentration was 200 µM. Error bars represent standard deviations (*n* = 4).

The different emulsions were incubated for 8 days, and primary (conjugated diene hydroperoxides) and secondary (pAV) lipid oxidation markers were recorded (Figure 4). After one day, conjugated diene formation was low and remained below 15 mmol/kg oil in all emulsions, except for the free iron emulsion. During incubation, the conjugated diene formation in the free iron and iron-loaded bead emulsions showed the same trend and ultimately reached values of approximately 145–165 mmol/kg oil. The empty bead emulsion followed the same trend as the control emulsion, with levels remaining below 16 mmol/kg for 3 days and increasing to around 120 mmol/ kg oil after 8 days. The presence of iron thus seems to enhance the formation of primary oxidation markers, as expected. 

There was a clear difference in the formation kinetics of secondary oxidation markers (pAV) in the free iron emulsion compared to the other emulsions. The free iron emulsion had a pAV of nine (AU) after 1 day, which increased continuously up to 34. The pAV in the emulsion containing iron-loaded beads slowly increased to approximately 10 and remained below seven in the other two. This, therefore, shows that even though primary lipid oxidation products formed in a quite similar manner for both iron-containing emulsions, this was not the case for the secondary lipid oxidation products. This indicates that encapsulating ferrous sulfate in alginate beads only has a minor effect on the *formation* of hydroperoxides but can effectively delay their *decomposition* into secondary lipid oxidation products. This can be because the formation of hydroperoxides occurs through an autocatalytic radical chain reaction, where peroxyl radicals (LOO^●^), formed from O_2_ addition to an alkyl radical (L^●^), abstract hydrogen atoms from other unsaturated lipids (L’H) to form hydroperoxides (LOOH). Conversely, the decomposition of hydroperoxides in alkoxyl radicals (LO^●^) is a fast reaction promoted by low valence transition metals such as ferrous iron, which themselves become oxidized through the process [32].

From these results, it is clear that the encapsulation of iron in alginate beads increased the oxidative stability of the emulsion, which is important for the sensory quality of food, which is linked to the formation of secondary oxidation products. In earlier work [20], we also found that embedding iron in an alginate matrix improved the chemical stability and sensory quality of cocoa-containing nut paste compared to free iron and liposomal iron. In the next sections, we try to elucidate how the pro-oxidant activity of iron is decreased, although not totally prevented, by encapsulation in alginate beads. For this, it seems relevant to take inspiration from examples of environmental engineering in which iron-loaded alginate beads have been used to initiate Fenton-type oxidation to remove malodorous compounds [33] or pharmaceutical pollutants [34], while allowing for a facile removal post-reaction due to the physical sequestration of iron. 

### 3.4. Estimation of the Reactive Iron Fraction in Iron-Loaded Beads

**Figure 5:**
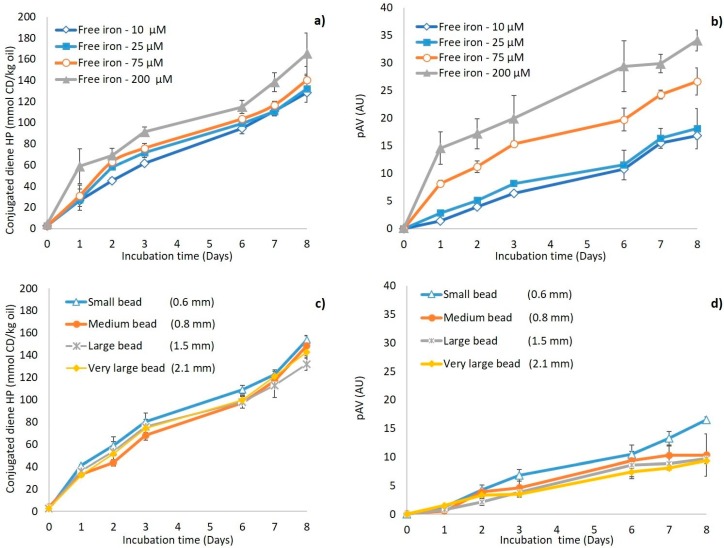
Conjugated diene (CD) hydroperoxide concentration and para-anisidine values (pAV) in O/W emulsions: (**a**,**b**) with various concentrations of free iron (10 µM (◊), 25 µM (■), 75 µM (○), and 200 µM (▲)); and (**c**,**d**) with iron-loaded alginate beads of various size (small bead (∆), medium bead (●), large bead (Ж), and very large bead (♦)). Error bars represent standard deviations (*n* = 4).

The previous results imply that encapsulating ferrous surface in alginate beads reduces its pro-oxidant activity, but that a fraction of iron is still reactive, which could be the fraction located close to the bead surface. In order to test this surface iron hypothesis, and to estimate how large this reactive fraction is, we measured lipid oxidation in emulsions containing different amounts of free iron, and in emulsions fortified with iron-loaded beads with different bead sizes (which is expected to correlate with differences in available surface iron). The formation of primary and secondary lipid oxidation markers is shown in Figure 5a,b for emulsions with various concentrations of free iron, and in Figure 5c,d for emulsions containing iron-loaded beads with different sizes. Decreasing the free iron concentration from 200 to 10 µM resulted in a limited decrease in the formation of conjugated dienes (Figure 5a), but to a substantial reduction in the formation of secondary lipid oxidation products (Figure 5b). In line with the remarks made in the previous section, this suggests that the formation of hydroperoxides is less iron concentration dependent than their decomposition into secondary lipid oxidation products. This also shows that the fraction of iron that is still reactive in the iron-loaded alginate beads is low, since the pAV in the emulsion incubated with iron-loaded alginate beads (Figure 4b) reached similar, or even slightly lower, values compared to the emulsion with the lowest free iron concentrations tested, i.e., 10 µM (Figure 5b). It is expected that reactive iron would be only that portion of the iron that is located on/very close to the surface of the beads, whereas the bead core-entrapped iron would not be reactive. This hypothesis implies that the fraction of reactive iron, and thus the pro-oxidant activity, would depend on the surface-to-volume ratio of the alginate beads, and thus simply on their size. We observed that emulsions containing large iron-loaded beads were seemingly less susceptible to oxidation than emulsions containing small ones (Figure 5c,d). From these results, it seems possible to roughly estimate the thickness of the layer that contains reactive iron at the surface of the alginate beads, assuming that the physical distribution of iron within the beads is homogeneous: for medium-sized beads (*d_43_* = 0.82 mm), if encapsulation in alginate beads reduces the concentration of reactive iron by approximately 20 times, this implies that the layer containing reactive iron represents approximately 5% of the bead volume, i.e., it is approximately 1.2 µm.

An analysis of the elemental composition of the alginate bead surface by energy-dispersive X-ray spectroscopy (EDX) is shown in Table 3. This technique can be used to map the elemental composition of samples with a depth resolution of up to a few µm, which is thus of the same order of magnitude as the expected thickness of the reactive layer, as determined earlier. This confirms that for iron-loaded alginate beads, some iron is present at the surface, with a weight percentage of ferrous iron of 3%. Furthermore, it is good to mention that the amount of calcium is slightly lower in iron-loaded beads compared to empty beads, which supports the notion that both cations bind to the alginate and may compete for electrostatic bonds with calcium within the alginate matrix [25]. 

### 3.5. Effect of a Second Alginate Layer on the Pro-Oxidant Activity of Iron

**Figure 6:**
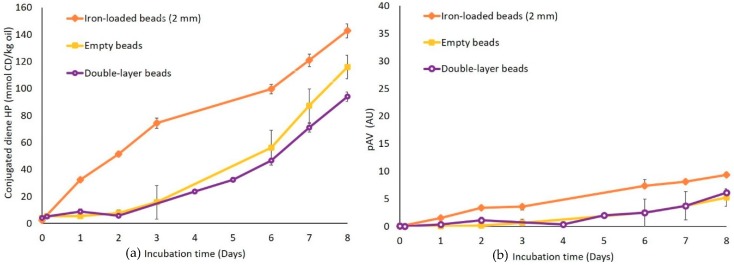
(**a**) Conjugated diene (CD) hydroperoxide (**b**) and para-anisidine values (pAV) in O/W emulsions with iron-loaded alginate beads (●), empty alginate beads (■), and double-layer alginate beads (○). All emulsions were incubated at 25 °C under slow rotative agitation. For the two emulsions containing iron, the FeSO_4_ concentration was 200 µM. Error bars represent standard deviations (*n* = 4).

In an attempt to suppress the pro-oxidant activity of surface-bound iron in alginate beads, a second alginate layer (typically in the order of 0.5 mm) was applied on very large iron-loaded alginate beads that are relatively easy to handle (Figure S1), which were next applied in emulsions for 8 days of incubation (Figure 6). The formation of both primary and secondary lipid oxidation markers was substantially reduced compared to the emulsion containing standard iron-loaded beads and was actually similar to what was obtained for reference emulsions containing empty beads. This implies that the second alginate layer completely prevents iron-promoted lipid oxidation, therefore proving to be an efficient encapsulation strategy. 

The results show that the localization and chemical availability of soluble iron has a great influence on the oxidative stability of emulsions. Encapsulating ferrous sulfate into alginate beads leads to the considerable improvement of the oxidative stability of ion-fortified emulsions, compared to the use of free iron, especially in relation to the formation of secondary oxidation markers. The most efficient inhibition of the pro-oxidant activity of iron was obtained when preventing contact between the surrounding emulsion matrix and the iron-containing surface of the beads, by application of a second alginate layer. Such advanced encapsulates seem highly effective for delivering soluble iron without compromising the chemical stability of the food systems. 

## 4. Conclusions

Ferrous sulfate can be encapsulated at high efficiency (>80%) in an alginate gel matrix. Such iron-loaded alginate beads showed high physical stability and improved the oxidative stability of emulsions compared to emulsions containing free iron. The presence of some reactive iron at the bead surface was associated with a residual pro-oxidant activity, which could be completely inhibited by applying a second alginate layer. The developed double-layer iron-loaded alginate beads show interesting potential for the fortification of foods without causing negative side effects, such as lipid oxidation.

## Figures and Tables

**Table 1:** Average diameter (*d[4,3]*) and specific surface area of iron-loaded alginate beads produced with various air pressures. Reported values are the average ± standard deviation of 20 measurements performed for two independently prepared batches.

Sample Name	*d[4,3]* (mm)	Specific Surface Area (m^2^/kg Bead)
Very large bead (0.2 bar)	2.05 ± 0.11 ^a^	2.9
Large bead (1 bar)	1.51 ± 0.04 ^b^	4.0
Medium bead (2 bar)	0.82 ± 0.01 ^c^	7.3
Small bead (3 bar)	0.62 ± 0.02 ^d^	9.7
Empty medium bead	0.76 ± 0.02 ^e^	7.9

Mean ± SD, *n* = 6. Values with different letters in column are significantly different according to Tukey’s b test (*p* < 0.05).

**Table 2:** Free fatty acid values in stripped rapeseed oil, and in emulsions. Values are the average ± standard deviation of three measurements performed on two independently prepared batches. Values marked with different letters are significantly different according to Tukey’s b test (*p* < 0.05).

Sample	mg KOH/g Oil
Stripped oil	0.02 ± 0.005 ^a^
Control emulsion (0th day)	0.06 ± 0.008 ^b^
Control emulsion (8th day)	0.65 ± 0.011 ^c^
Free iron emulsion (8th day)	1.02 ± 0.006 ^d^
Iron-loaded bead emulsion (8th day)	0.94 ± 0.009 ^e^

**Table 3:** Surface composition (wt%) of iron-loaded and empty alginate beads determined by energy dispersive X-ray spectroscopy (EDX).

Element	O	Na	S	Cl	Ca	Fe
Iron-loaded beads	59.54	0.88	0.48	17.61	18.51	2.98
Empty beads	59.30	1.00	-	19.86	19.85	-

## Supplementary Materials

The following are available online at https://www.mdpi.com/2304-8158/8/9/361/s1, Figure S1: Schematic representation of the production of regular and double-layer iron-loaded alginate beads, Figure S2: Particle size distributions of emulsions, freshly prepared and after 8 days of incubation.
