# Supplementary material for: Lipid Oxidation in Emulsions Fortified with Iron-Loaded Alginate Beads
Source: Foods. 2019 Aug 24;8(9):361. doi: 10.3390/foods8090361 (PMC6769985; doi:10.3390/foods8090361)
Supplement: Supplementary file 1 [file foods-08-00361-s001.pdf]

Supplementary Materials:

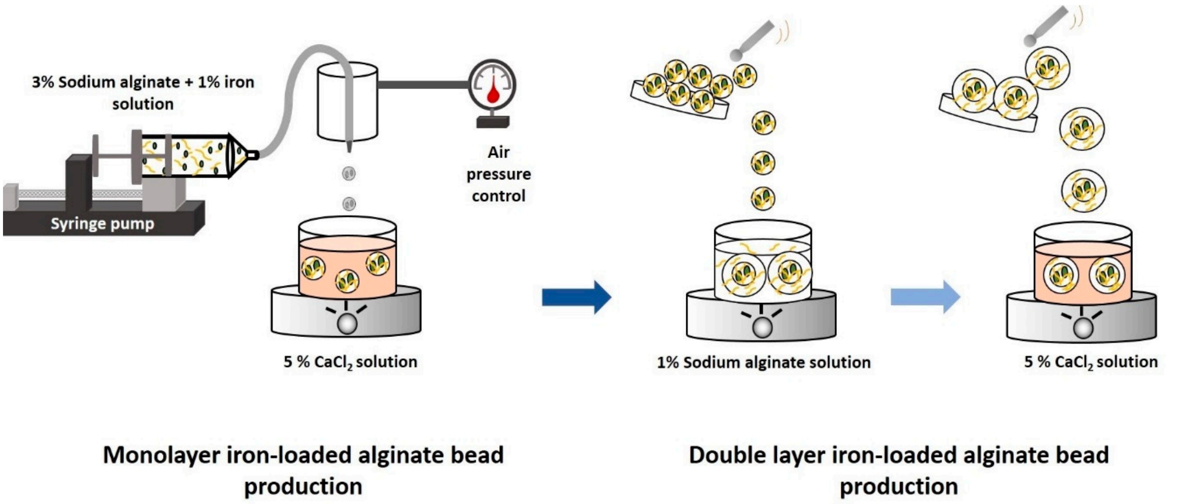

Figure S1 : Schematic representation of the production of regular and double-layer iron-loaded alginate beads. More details about the process settings can be found in the materials and methods section; the size of the beads is given in Table 1. The image is not to scale; the thickness of the second alginate layer is exaggerated.

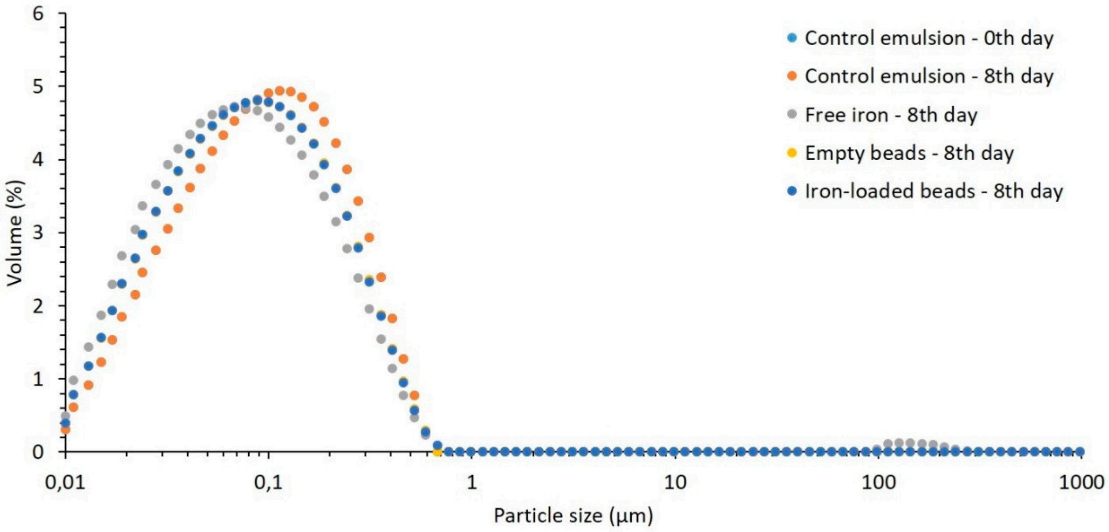

Figure S2 : Particle size distributions of emulsions, freshly prepared and after 8 days of incubation. Please note that the size distributions of the control emulsions at day 0 and 8, as well as those of the emulsions with empty beads or iron-loaded beads at day 8, overlap.
